# Effect of Sishen Pill on Memory T Cells From Experimental Colitis Induced by Dextran Sulfate Sodium

**DOI:** 10.3389/fphar.2020.00908

**Published:** 2020-07-02

**Authors:** Wei Ge, Hai-Yan Wang, Hai-Mei Zhao, Xue-Ke Liu, You-Bao Zhong, Jian Long, Zheng-Yun Zuo, Duan-Yong Liu

**Affiliations:** ^1^ Proctology Department, Affiliated Hospital of Jiangxi University of Traditional Chinese Medicine, Nanchang, China; ^2^ Party and School Office, Jiangxi University of Traditional Chinese Medicine, Nanchang, China; ^3^ College of Traditional Chinese Medicine, Jiangxi University of Traditional Chinese Medicine, Nanchang, China; ^4^ Department of Postgraduate, Jiangxi University of Traditional Chinese Medicine, Nanchang, China; ^5^ Science and Technology College, Jiangxi University of Traditional Chinese Medicine, Nanchang, China; ^6^ Pharmacology Office, Key Laboratory of Pharmacology of Traditional Chinese Medicine in Jiangxi, Nanchang, China

**Keywords:** Sishen pill, experimental colitis, memory T cells, P13K/Akt, mechanism of action

## Abstract

Immune memory has a protective effect on the human body, but abnormal immune memory is closely related to the occurrence and development of autoimmune diseases including inflammatory bowel disease (IBD). Sishen Pill (SSP) is a classic prescription of traditional Chinese medicine, which is often used to treat chronic colitis, but it is not clear whether SSP can alleviate experimental colitis by remodeling immune memory. In the present study, the therapeutic effect of SSP on chronic colitis induced by dextran sulfate sodium (DSS) was evaluated by colonic length, colonic weight index, macroscopic and microscopic scores, and pathological observation. The cytokine levels were tested by enzyme-linked immunosorbent assay (ELISA); the percentages of central memory T (Tcm) and effector memory T (Tem) cells were analyze\d by flow cytometry; and activation of phosphoinositide 3-kinase (PI3K)/Akt signaling proteins was measured by western blotting. After 7-days' treatment, SSP alleviated DSS-induced colitis, which was demonstrated by decreased colonic weight index, colonic weight, histopathological injury scores, restored colonic length, gradual recovery of colonic mucosa, and lower levels of interleukin (IL)-2, IL-7, IL-12, and IL-15, while SSP increased IL-10 expression. SSP obviously regulated the quantity and subpopulation of Tcm and Tem cells. Furthermore, SSP markedly inhibited activation of PI3K, Akt, phospho-Akt, Id2, T-bet, forkhead box O3a, Noxa, and C-myc proteins in the PI3K/Akt signaling pathway and activated Rictor, Raptor, tuberous sclerosis complex (TSC)1, TSC2, phospho-AMP-activated kinase (AMPK)-*α*, AMPK-*α*, eukaryotic translation initiation factor 4E-binding protein 2, kinesin family member 2a, and 70-kDa ribosomal protein S6 kinase. These results indicate that SSP effectively controls Tem cells in the peripheral blood to relieve experimental colitis induced by DSS, which were potentially related with inhibiting the PI3K/Akt signaling pathway.

## Introduction

Inflammatory bowel disease (IBD) is an immune-mediated chronic nonspecific intestinal inflammatory disease, including ulcerative colitis (UC) and Crohn's disease (CD), with the characteristics of alternating remission and recurrence (Xavier and Podolsky, 2007). Although the exact etiology and pathogenesis of IBD are not clear, more studies have shown that susceptibility genes are induced by environmental factors to produce inappropriate immune responses with symbiotic microorganisms (Zhang et al., 2017). The inappropriate immune response may be related to the abnormal level of memory T cells that remain in the intestinal tract of IBD patients for a long time and make intestinal inflammation difficult to alleviate and easy to recur (Kanai et al., 2009; Gattinoni et al., 2011). n IBD, memory T cells are intermittently reactivated in secondary lymphoid organs and thereafter return to inflammatory tissues (Rosen et al., 2003). These memory T cells can survive for a long period, and they provide the basis for long-term immunological memory. As a major subpopulation of effector memory T (Tem) cells, a substantial proportion of colitogenic CD4^+^ central memory T (Tcm) cells preferentially reside in colitic mice (Nemoto et al., 2007; Nemoto et al., 2009). Fujii et al. (2006) have found that FTY720 inhibits the level of CD4^+^ Tem and CD4^+^ Tcm cells to prevent the development of colitis induced by the adoptive transfer of *lamina propria* (LP) colitogenic CD4^+^ Tem cells. These studies have indicated potential and effective tactics to prevent the onset and development of autoimmune diseases, including IBD by regulating the function and status of memory T cells.

Phosphoinositide 3-kinase (PI3K)/Akt signaling plays an important role in a variety of cell signaling pathways, cell cycle progression, and cell growth including T follicular helper cells (Way et al., 2016). Under the participation of many inflammatory cytokines including interleukin (IL)-2 and IL-7, PI3K/Akt signal activation can regulate the differentiation and maintenance of immune memory T cells. Its downstream effector factors mammalian target of rapamycin (mTOR) and forkhead box (FOXO) can mediate the transformation of T lymphocytes from synthetic metabolism of effector cells to catabolic metabolism of memory cells and are beneficial for differentiation of effector cells into memory CD8^+^ T cells (Sullivan et al., 2012; Kim and Suresh, 2013). Therefore, we believe that the PI3K/Akt signaling pathway should be a central target to further regulate the differentiation and transformation of memory T cell subpopulations to treat IBD.

As a classic prescription of traditional Chinese medicine, Sishen Pill (SSP) has been treating chronic enteritis in China for thousands of years. A clinical study of 204 patients with IBD found that the total effective rate of SSP was 75.98%, and the recurrence rate within 6 months was only 8.1%, while the recurrence rate with sulfasalazine was 23.3% (Lu et al., 2011). Although there is no direct evidence that SSP treats IBD by regulating immune memory, we found that SSP relieved experimental colitis by inhibiting the PI3K/Akt signaling pathway and regulating the balance between proinflammatory and anti-inflammatory cytokines (Liu et al., 2012; Zhao et al., 2013; Liu et al., 2015). Therefore, in the present study, memory T cells and the PI3K/Akt signaling pathway were considered as two candidate observation points to explore the mechanism of action of SSP alleviation and treatment of IBD.

## Materials and Methods

### Mice

Male BALB/c mice aged 9–12 weeks, weighing 20–22 g, were purchased from the Hunan Silaike Jingda Experimental Animal Co. Ltd. (Changsha, China) (Animal Certificate Number SCXK 2006-0008). All animals were housed at the Jiangxi University of Traditional Chinese Medicine animal facility in specific-pathogen-free conditions. The present protocol (Permit Number: JZ2018-120) was approved by the Institutional Animal Care and Use Committee of Jiangxi University of Traditional Chinese Medicine. All animals were acclimatized to the animal center conditions for 3 days before the experimental studies were performed. Forty mice were divided into two groups: 10 in the normal group and 30 with experimental colitis induced by dextran sulfate sodium (DSS). After colitis induction, the mice were randomly distributed into three groups: DSS: untreated with DSS-induced colitis; DSS + SSP: DSS-induced colitis treated with SSP; and DSS + 5-ASA: DSS-induced colitis treated with 5-ASA (mesalazine).

### Drugs

SSP (batch number 17080051) was purchased from Tongrentang Natural Medicine Co. Ltd. (Beijing, China), was composed of *Euodia rutaecarpa* (Juss.) Benth., *Schisandra chinensis* (Turcz.) Baill, *Psoralea corylifolia L., Myristica fragrans* Houtt*., Ziziphus jujuba* Mill*. and Zingiber officinale* Rosc. which were prepared into pills according to the dose ratio (respectively 100, 200, 400, 200, 200 and 200 g, ratio: *1:2:4:2:2:2*). The previous study of Zhang et al. (2018) measured the main active ingredients of SSP by HPLC-ESI-MS/MS, which include isopsoralen (1293.7 μg/g), schizandrin (258.0 μg/g), *γ*-schizandrin (131.5 μg/g), psoralen (131.08 μg/g), and deoxyschizandrin (72.6 μg/g), and so on (as shown in **Table 1**). DSS (molecular weight: 36,000–50,000 kDa) was obtained from MP Biomedicals (Santa Ana, CA, USA) and 5-ASA (Sunflower Pharma, Jiamusi, China).

**Table 1:** The quantitative determination of nine components in SSP by HPLC-ESI-MS/MS.

NO.	Components	Linear Ranges (ng/ml)	Average Recoveries	Quantitative Determination (μg·g^−1^)
1	**Deoxyschizandrin**	8.50–850.00 (*r *= 0.9997)	98.3% (RSD = 2.21%)	72.6
2	***γ*-schizandrin**	1.32–132.00 (*r *= 0.9974)	100.3% (RSD = 1.78%)	131.5
3	**Schizandrin**	9.60–960.00 (*r *= 0.9998)	99.2% (RSD = 2.19%)	258.0
4	**schizandrol B**	12.00–1,200.00 (*r *= 0.9993)	100.4% (RSD = 2.23%)	71.2
5	**schisantherin A**	11.50–1,150.00 (*r *= 0.9979)	99.1% (RSD = 2.18%)	25.1
6	**psoralen**	21.70–2,170.00 (*r *= 0.9997)	97.7% (RSD = 3.03%)	1,310.8
7	**isopsoralen**	23.80–2,380.00 (*r *= 0.9996)	99.0% (RSD = 2.51%)	1,293.7
8	**evodiamine**	10.70–1,070.00 (*r *= 0.9995)	98.9% (RSD = 2.72%)	22.2
9	**rutaecarpine**	8.54–854.00 (*r *= 0.9980)	100.3% (RSD = 2.10%)	24.0

The quantitative determination of SSP was finished by Zhang et al. (2018) via HPLC-ESI-MS/MS. The above data were from the paper written by Zhang et al. and published in Chinese Traditional and Herbal Drugs in 2018. In this study, the SSP is purchased from Tongrentang Natural Medicine Co. Ltd. (Beijing, China), which batch number is 17080051 and is the same with our present study. All results have shown that the quality of SSP (17080051) is definite and controlled.

### DSS-Induced Experimental Colitis

To establish experimental colitis, mice were given 3% (w/v) DSS in their drinking water for 7 days. Mice were weighed once daily (09:00) to determine body weight change and monitored daily for diarrhea, hematochezia, hunched posture, weight loss, and hair loss.

### Therapeutic Protocols

On day 8, the DSS + SSP group was treated with 2.5 g/kg SSP dissolved in physiological saline by oral gavage for 7 days; the DSS + 5-ASA group was treated with 300 mg/kg mesalazine by oral gavage for 7 days; and the DSS and normal groups were treated with the same volume of physiological saline by oral gavage for 7 days. On day 15, all animals were sacrificed after anesthesia with sodium pentobarbital.

### Macroscopic Observation

The colons were removed quickly from the mice after death, and the length was measured. The colon was then washed and opened longitudinally along the mesentery. The index of colonic weight (n = 10) was calculated according to colonic weight/body weight × 100%. The macroscopic assessment was carried out with reference to Butzner et al. (1996), and the histological injury score was established according to the criteria reported by Schmidt et al. (2010). The total score included inflammatory cell infiltration and tissue injury.

### Histological Analysis

The colonic tissues were collected and fixed in 4% polyformaldehyde solution for 7 days and then dehydrated and embedded in paraffin. Five-micrometer-thick sections were cut and stained with hematoxylin and eosin (H&E), as described in previous study (Cribiu et al., 2018; Cribiu et al., 2019) and observed under an Olympus microscope (scanning resolution 50,000 pixels per inch, 0.5 µm per pixel with 10× objective and 2.5 µm per pixel when scanning at 4×). Histopathological evaluation of colonic tissue (n = 10) was performed according to the standards reported by Mahler et al. (1998).

### ELISA

To test the yields of IL-2, IL-10, IL-7, IL-12, and IL-15 in the colonic tissue by ELISA, portions of the colonic tissue (n = 10) were homogenized in 300 µl RIPA Buffer (Cell Signaling Technology, Danvers, MA, USA) and incubated at slow rotation (4°C, 30 min) and then centrifuged at 4°C for 30 min at 13,000 rpm to obtain tissue supernatant. The production of IL-2, IL-7, IL-10, IL-12, and IL-15 in colonic tissues was measured by commercial ELISA kits (eBioscience, San Diego, CA, USA), and then absorbance at 450 nm was read by an enzyme labeling instrument (Bio-Rad, Hemel Hempstead, UK).

### Western Blotting

The supernatant of colonic tissues used for western blotting was prepared as described for ELISA. The concentration of protein was determined by the classical BCA protein determination method (Beyotime, Nanjing China). The same amount of protein was separated by 10% SDS-PAGE and transferred to polyvinylidene fluoride membranes (Millipore, Billerica, MA, USA). After sealing with 5% skimmed milk, the membranes were incubated with primary antibodies overnight at 4°C. The antibodies were anti-GAPDH (1:3,000), phosphoinositide 3-kinase (PI3K) (1:2,000), Akt (1:2,000), phospho (p)-Akt (1:1,500), Id2 (1:1,000), T-bet (1:2,000), FOXO3a (1:1,000), Noxa (1:2,000), C-myc (1:2,000), Rictor (1:2,000), Raptor (1:2,000), tuberous sclerosis complex (TSC)1 (1:1,000), TSC2 (1:2,000), p-AMP-activated kinase α (1:2000), AMPKα (1:2,000), eukaryotic translation initiation factor 4E-binding protein (4E-BP)2 (1:1,000), kinesin family member (Kif)2a (1:1,500), and 70-kDa ribosomal protein S6 kinase (p70S6K) (1:2,000). All primary antibodies were purchased from Abcam (Cambridge, UK). Then the membranes were washed with tris buffered saline tween (TBST) and incubated with the secondary antibody (1:2,000–1:4,000) (Abcam) at 37°C for 1 h. After washing with TBST again, the labeled protein bands were scanned with HP Scanjet 5500 (Hewlett Packard France, Les Ullis, France).

### Flow Cytometry

The numbers of CD45RA^−^CD62L^+^CCR7^+^ T (Tcm) cells, CD45RA^−^CD62L^−^CCR7^+^ (Tem) T cells and their CD4^+^ and CD8^+^ subsets in the peripheral blood were detected by flow cytometry. For isolation of peripheral lymphocytes, 500 μl peripheral blood was collected from each mouse and lysed by treatment with 1 ml lysing Solution (BD Biosciences, Franklin Lakes, NJ, USA) to clear red blood cells. The obtained lymphocytes were incubated with fluorescence-conjugated monoclonal antibodies in staining buffer. Eight-color flow cytometry analysis (*n* = 10) was performed on a FACS Calibur device (Becton-Dickinson, Mountain View, CA). Memory T cells were identified as an CCR7^+^ lineage^+^ (CD45RA^+^, CD62L^+^) population to differ the Tm and Tem cells, and within this group, the CD4^+^ and CD8^+^ population was assessed too. In these cell suspensions, the following steps were performed. To 100 μl anticoagulant, added 100 μl RPMI 1640 medium and 1 ml hemolysin, incubated for 15 min; centrifuged at 300 g for 5 min, removed supernatant; added 1 ml stain buffer (554656) and rinsed cells twice; centrifuged at 300 g for 5 min, discarded supernatant; resuspended cells with 100 μl stain buffer; added 1 μg FC blocking sealant (553141), incubated at 4° for 8 min; added primary antibodies, incubated for 15 min at room temperature; washed twice with 1 ml stain buffer, discarded supernatant; and added 500 μl stain buffer to resuspend cells. Samples were detected by FACSCanto II flow cytometry (BD Biosciences, Franklin Lakes, NJ, USA). The following mAbs were used: APC-H7 rat anti-mouse CD4 (1:200), Alexa Fluor 647 rat anti-mouse CCR7 (1:100), PE rat anti-mouse CD45RA (1:100), PE-CY7 rat anti-mouse CD62L (1:100), and FITC rat anti-mouse CD8 (1:100), (BD Biosciences). Limits for the quadrant markers were set based on negative populations and isotype controls. The data were analyzed by FlowJo software 10 (TreeStar, Ashland, OR, USA), and the inactive cells were excluded by gating.

### Statistical Analysis

All parameters were expressed as mean ± SEM. All statistical analyses were performed using GraphPad Prism version 8.0 (San Diego, CA, USA). Significance was determined using one-way analysis of variance followed by the Tukey test for multiple comparisons. p < 0.05 was considered significant.

## Results

### SSP Ameliorated DSS-Induced Colitis

**Figure 1:**
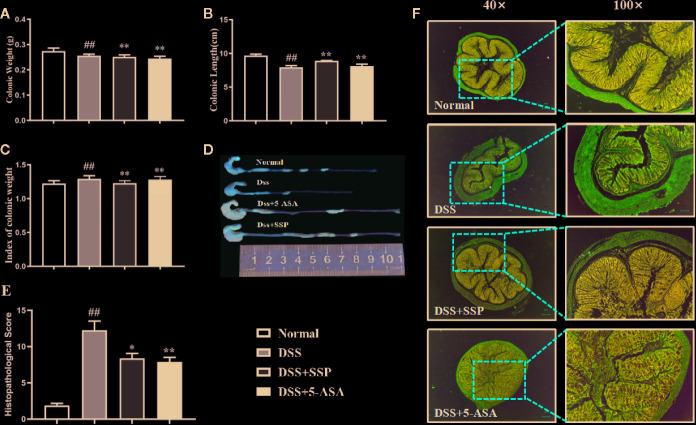
Therapeutic evaluation of SSP amelioration of DSS-induced colitis in mice. **(A)** Colonic weight. **(B)** Colonic length. **(C)** Colonic weight index. **(D)** Changes in colonic length by naked eye. **(E)** Histopathological score. **(F)** H&E staining. Data are presented as mean ± SEM (n = 10). ^##^p < 0.01 *versus* normal group, *p < 0.05 and **p < 0.01 *versus* DSS group.

DSS-induced colitis is a classic animal model to develop new drugs for treatment of IBD, whose clinical and pathological characteristics are similar to those of human IBD. As expected in our study, the characteristics of diarrhea, hematochezia, hunched posture and weight loss in DSS-induced and untreated mice were similar to those observed in human IBD patients. Colonic weight (**Figure 1A**) and colonic weight index (**Figure 1C**) of colitis mice in the DSS group were higher, and the colonic length (**Figures 1B, D**) was shorter than in the normal group. Pathological observation (**Figure 1F**) found abundant inflammatory cell infiltration, hyperemia, edema, crypt disappearance and malformation, thickening colonic wall, and ulcer formation in the colitis mice without treatment. The histopathological score (**Figure 1E**) was increased in colitis mice in the DSS group when compared with the normal mice. Significantly, after 7-days' treatment with SSP and 5-ASA, the histological colonic injury (**Figure 1F**) was improved, with fewer inflammatory cells in the colonic mucosa and submucosa, intact mucosa, and smaller and fewer ulcers. The injury score (**Figure 1E**) was decreased, the colonic length (**Figures 1B, D**) was restored, and the colonic weight index (**Figure 1C**) was reduced when they were compared with colitis mice in the DSS group. These results indicated that SSP effectively ameliorated DSS-induced colitis.

### SSP Regulated Tcm Cell Level

**Figure 2:**
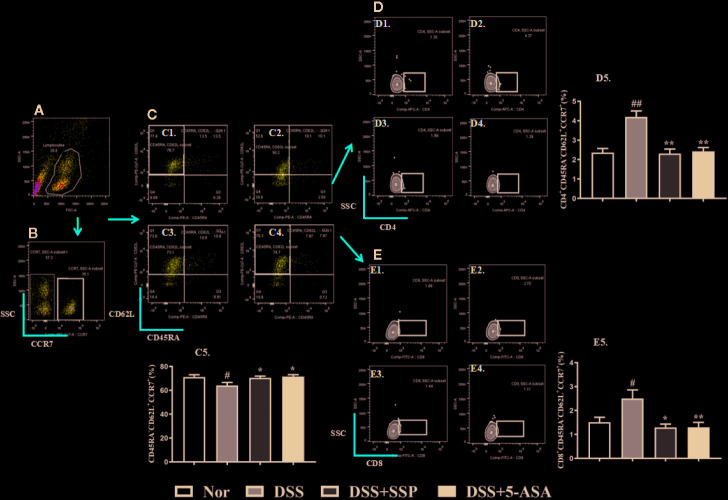
SSP regulated Tcm cells in colitis mice. **(A)** Total lymphocytes in peripheral blood. **(B)** CCR7^+^ lymphocytes were measured by flow cytometry. **(C)** CD45R- and CD62L-labeled lymphocytes; cells in the left upper side are Tcm cells. C1: Tcm cells in normal group; C2: Tcm cells in DSS group; C3: Tcm cells in DSS + SSP group; C4: Tcm cells in DSS + 5-ASA group; C5: statistical analysis of Tcm cells in the four groups. **(D)** CD4^+^ Tcm cell distribution analyzed by flow cytometry. D1: CD4^+^ Tcm cells in normal group; D2: CD4^+^ Tcm cells in DSS group; D3: CD4^+^ Tcm cells in DSS + SSP group; D4: CD4^+^ Tcm cells in DSS + 5-ASA group; D5: statistical analysis of CD4^+^ Tcm cells in the four groups. **(E)** CD8^+^ Tcm cell distribution analyzed by flow cytometry. E1: CD8^+^ Tcm cells in normal group; E2: CD8^+^ Tcm cells in DSS group; E3: CD8^+^ Tcm cells in DSS + SSP group; E4: CD8^+^ Tcm cells in DSS + 5-ASA group; E5: statistical analysis of CD8^+^ Tcm cells in the four groups. Data are presented as mean ± SEM (n = 10). ^#^p < 0.05 and ^##^p < 0.01 *versus* normal group; *p < 0.05 and **p < 0.01 *versus* DSS group.

Lymph node homing receptor CD62L and chemokine CC receptor (CCR)7 (**Figures 2A, B**) were used to identify and count memory T cells. Tcm cells are an important component of memory T cells that express CD62L and CCR7 but not CD45RA. Tcm cells can return to the secondary lymphoid organs and proliferate stably and differentiate into effector T cells under antigen stimulation. In the present study, the CD45RA^−^CD62L^+^CCR7^+^ cells were the Tcm cells (**Figures 2C1–4**). The number of CD45RA^−^CD62L^+^CCR7^+^ cells (**Figures2C1–5**) was dramatically reduced in the untreated colitis mice in contrast to the normal mice. This suggested that the lower number of Tcm cells was one of the main characteristics of DSS-induced colitis in our study. Compared with the DSS group, the number of Tcm cells increased significantly in the normal, DSS + SSP, and DSS + 5-ASA groups. As the two subsets of Tcm cells, CD4^+^CD45RA^−^CD62L^+^CCR7^+^ (**Figures 2D1–5**) and CD8^+^CD45RA^−^CD62L^+^CCR7^+^ (**Figures 2E1–5**) cells were downregulated after colitis mice were treated by SSP and 5-ASA for 7 days. SSP controlled the number of Tcm cells and their subsets in DSS-induced colitis.

### SSP Regulated Tem Cell Level

**Figure 3:**
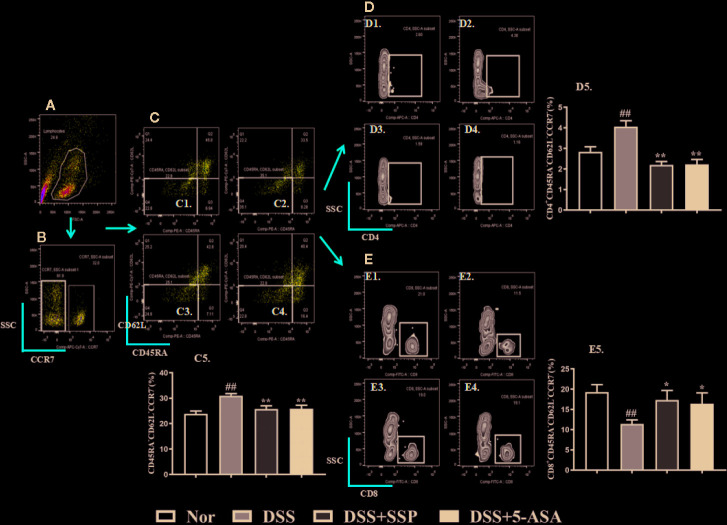
SSP regulated Tem cells in colitis mice. **(A)** Total lymphocytes in peripheral blood. **(B)** CCR7^−^ lymphocytes measured by flow cytometry. **(C)** Double-negative CD45R and CD62L lymphocytes. Cells in the lower left side are Tem cells. C1: Tem cells in normal group; C2: Tem cells in DSS group; C3: Tem cells in DSS + SSP group; C4: Tem cells in DSS + 5-ASA group; C5: statistical analysis of Tem cells in the four groups. **(D)** CD4^+^ Tem cell distribution analyzed by flow cytometry. D1: CD4^+^ Tem cells in normal group; D2: CD4^+^ Tem cells in DSS group; D3: CD4^+^ Tem cells in DSS + SSP group; D4>: CD4^+^ Tem cells in DSS + 5-ASA group; D5: statistical analysis of CD4^+^ Tem cells in the four groups. **(E)** CD8^+^ Tem cell distribution analyzed by flow cytometry. E1: CD8^+^ Tem cells in normal group; E2: CD8^+^ Tem cells in DSS group; E3: CD8^+^ Tem cells in DSS + SSP group; E4: CD8^+^ Tem cells in DSS + 5-ASA group; E5: statistical analysis of CD8^+^ Tem cells in the four groups. Data are presented as mean ± SEM (n = 10). ^##^p < 0.01 *versus* normal group; *p < 0.05 and **p < 0.01 *versus* DSS group.

Tem cells can migrate to the site of peripheral inflammation and exert an immune effect, and they do not express CCR7 (**Figures 3A, B**), CD45RA,and CD62L on their surface. In the DSS group, the number of CD45RA^−^CD62L^−^CCR7^−^ Tem cells increased significantly (**Figures 3C1–5**); the number of CD4^+^ Tem cells showed a significant upward trend (**Figures 3D1–5**); and the total number of CD8^+^ Tem cells had a marked downward trend (**Figures 3E1–5**). After SSP and 5-ASA treatment for 7 days, the number of CD4^+^ Tem (**Figures 3D1–5**) was decreased, and the number of CD8^+^ Tem cells was increased (**Figures 3E1–5**), when compared with the number of cells in the DSS group. The results showed that SSP regulated Tem cell number and the balance of Tem subsets.

### SSP Regulated Inflammatory Factors' Expression in Colitis Mice

**Figure 4:**
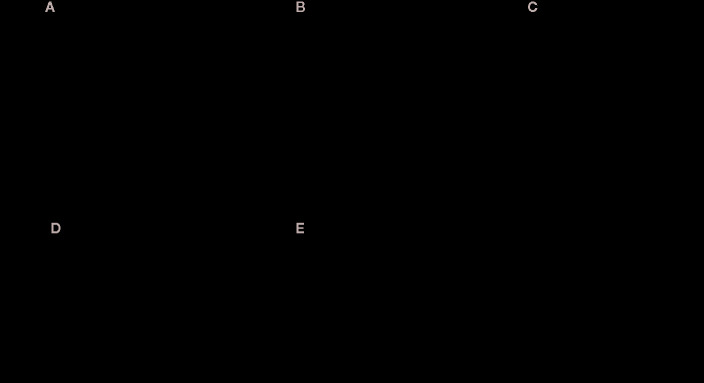
SSP inhibited IL-2, IL-7, IL-12, and IL-15 expression in DSS-induced colitis. **(A)** IL-2 expression; **(B)** IL-7 expression; **(C)** IL-10 expression; **(D)** IL-12 expression; **(E)** IL-15 expression. Data are presented as mean ± SEM (n = 10). ^#^p < 0.05 and ^##^p < 0.01 *versus* normal group, *p < 0.05 and **p < 0.01 *versus* DSS group.

Abnormal expression of inflammatory cytokines is an important characteristic of immunological injury in IBD, including IL-2, IL-7, IL-10, IL-12, and IL-15. Subsequently, these excessive cytokines induce the transformation and migration of Tcm and Tem. The secretion of IL-2 (**Figure 4A**), IL-7 (**Figure 4B**), IL-12 (**Figure 4D**), and IL-15 (**Figure 4E**) in colonic tissues from colitis mice in the DSS group was higher than that in the normal, DSS + SSP, and DSS + 5-ASA groups. While the IL-10 (**Figure 4C**) expression in DSS-induced colitis mice without treatment was lower than that in the normal mice and colitis mice treated with SSP or 5-ASA. The results suggested that SSP regulated the balance of proinflammatory and anti-inflammatory cytokines in colitis mice.

### SSP Controlled Activation of PI3K/Akt Pathway in Colitis Mice

**Figure 5:**
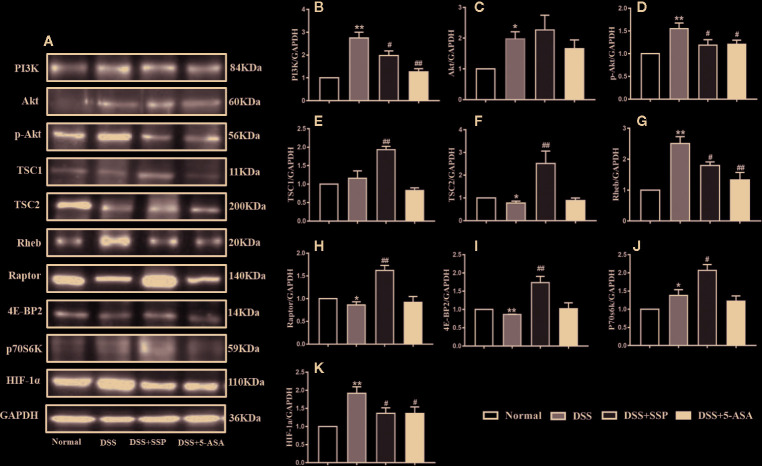
Activation PI3K/Akt signaling pathway in mice with DSS-induced colitis (I). After 7 days' administration of SSP, expression of kinases and proteins related to PI3K/Akt signaling transduction in colon was detected by western blotting to determine whether the PI3K/Akt signaling pathway was activated. **(A)** Western blotting of major proteins in the PI3K/Akt signaling pathway, such as PI3K, Akt, p-Akt, TSC1, TSC2, Rheb, Raptor, 4E-BP2, p70S6K and HIF-1*α*. **(B)** Quantitative analysis of PI3K; **(C)** Quantitative analysis of Akt; **(D)** Quantitative analysis of p-Akt; **(E)** Quantitative analysis of TSC1; **(F)** Quantitative analysis of TSC2; **(G)** Quantitative analysis of Rheb; **(H)** Quantitative analysis of Raptor; **(I)** Quantitative analysis of 4E-BP2; **(J)** Quantitative analysis of p70S6K; **(K)** Quantitative analysis of HIF-1*α*. *p < 0.05 and **p < 0.01 versus Normal group; ^#^p < 0.05 and ^##^p < 0.01 versus DSS group.

**Figure 6:**
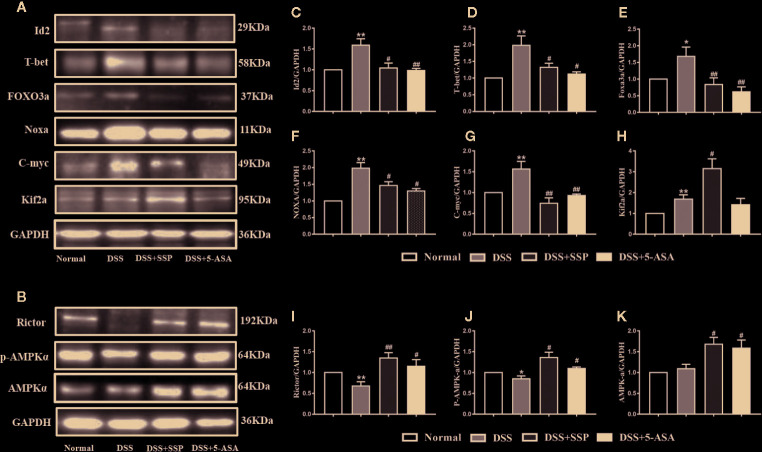
Activation PI3K/Akt signaling pathway in mice with DSS-induced colitis (II). **(A)** Western blotting of Id2, T-bet, FOXO3a, Noxa, C-myc, and Kif2a. **(B)** Western blotting of Rictor, AMPK*α*, and p-AMPK*α*. **(C)** Quantitative analysis of Id2; **(D)** Quantitative analysis of T-bet; **(E)** Quantitative analysis of FOXO3a; **(F)** Quantitative analysis of Noxa; **(G)** Quantitative analysis of C-myc; **(H)** Quantitative analysis of Kif2a; **(I)** Quantitative analysis of Rictor; **(J)** Quantitative analysis of p-AMPK*α*; **(K)** Quantitative analysis of AMPK*α*. *p < 0.05 and **p < 0.01 versus Normal group; ^#^p < 0.05 and ^##^p < 0.01 versus DSS group.

Western blotting showed that SSP had an effect on multiple proteins in the PI3K/Akt signaling pathway. As the central signaling event of the PI3K/Akt signaling pathway, Akt phosphorylation negatively regulates TSC1/TSC2 to maintain activation of Rheb and then enhance activation of mTOR complex (mTORC)1 to inhibit expression of 4E-BP2 by upregulating expression of p70S6K and hypoxia-inducible factor (HIF)-1*α*. Furthermore, it regulates synthesis of proteins and promotes cell growth and glycolysis. In the DSS group, expression of PI3K (**Figures 5A, B**), Akt (**Figure 5C**), p-Akt (**Figures 5A, D**), Rheb (**Figures 5A, G**), p70S6K (**Figures 5A, J**), and HIF-1*α* (**Figures 5A, K**) protein was significantly increased, while expression of TSC1 (**Figures 5A, E**), TSC2 (**Figures 5A, F**), and 4E-BP2 (**Figures 5A, I**) was decreased. It was suggested that the PI3K/Akt/mTORC1 signaling pathway was activated in DSS-induced colitis. In the DSS group, all the above results were obviously reversed after SSP treatment for 7 days. In the PI3K/Akt signaling pathway, we found that SSP activated the expression of PI3K/Akt signaling bypass proteins [Rictor (**Figures. 6B, I**), Raptor (**Figures 5A, H**), p-AMPK*α* (**Figures 6 B, J**), and AMPKα (**Figures 6B, K**)] and inhibited Id2 (**Figures 6A, C**), T-bet (**Figures 6A, D**), FOXO3a (**Figures 6A, E**), Noxa (**Figures 6 A, F**), and C-myc (**Figures 6A, G**) expression. The downstream proteins of the PI3K/Akt signaling pathway (4E-BP and p70S6K) were overtly elevated in the DSS + SSP and DSS + 5-ASA groups. The results revealed that SSP inhibited activation of the PI3K/Akt/mTORC1 signaling pathway in the colitis mice.

## Discussion

In this study, experimental colitis induced by DSS had obvious pathological characteristics of chronic colitis, such as mucosal erosion, formation of ulcer and granulation tissue, crypt structural changes, and inflammatory cell infiltration. Notably, after DSS-induced colitis mice were treated with SSP for 7 days, the colonic damage induced by DSS was alleviated or disappeared, which included decreased colonic weight index, downregulated histopathological score, and restored colonic length. The evidence indicated that SSP effectively attenuated DSS-induced colitis. The number of CD45RA^−^CD62L^−^CCR7^−^ Tem cells and CD4^+^ Tem cells increased significantly, while the number of CD8^+^ Tem cells decreased significantly in DSS-induced colitis. In addition, the overall number of CD45RA^−^CD62L^+^CCR7^+^ Tcm cells decreased, but the number of CD4^+^ and CD8^+^ Tcm cells increased significantly. These results suggest that there are significant changes in memory T cells and their subsets in DSS-induced colitis, and disorder of these immune memory cells may be an important reason for the persistent inflammatory state of IBD.

Immunologically, “memory” is now generally believed to be a phenomenon that occurs after Ags have been eliminated, which is defined as the ability of the immune system to respond with greater efficacy to a second encounter with the same pathogen as the self-antigens and commensal bacteria flora (Sallusto et al., 2004). So, the dysregulated homeostasis of memory-type pathogenic T cells plays a significant role in the pathogenesis of autoimmune disorders. Memory T cells are divided into Tcm cells and Tem cells according to whether they have the function of rapid response and the presence of corresponding homing receptors. Tcm cells can highly express CCR7 and CD62L, so they usually migrate to secondary lymphoid organs. They have strong potential for proliferation and can expand and differentiate into more effector T cells. However, Tem cells lose CCR7, which also determines that Tem cells can migrate to peripheral inflammation sites and play an effective role quickly (Pepper et al., 2011). In IBD, the Ags responsible for IBD are derived from commensal bacteria or self-antigens that can never be eliminated. As an additional explanation for the perpetuation of IBD, long-lived colitogenic memory Tcm or Tem cells are intermittently reactivated in secondary lymphoid organs and thereafter return to inflammatory tissues, such as the gut and colonic mucosa, which hinted that colitogenic “memory stem”-like Tcm cells are generated in the process of development and/or persistence of chronic colitis (Tamura et al., 2000; Oyama et al., 2005; Nemoto et al., 2009). Under the second Ags stimulation in the colonic tissue, Tcm cells lose the lymph node homing receptors CCR7 and CD62L (Sallusto et al., 2004), and transform into Tem cells, and form the colitogenic Tcm and/or Tem, and recirculate continuously between the blood, lymph, and colonic mucosa, to express high levels of inflammatory cytokines (as IL-2, IL-7, IL-12, and 1L-15 in **Figure 4**) that results in IBD. So, removing colitogenic memory T cell or regulating the proportion of the Tcm and Tem may have therapeutic benefit for the treatment of IBD (Tamura et al., 2000; Oyama et al., 2005; Nemoto et al., 2009).

In this study, the classic and pathological changes of DSS-induced colitis were synchronously observed with low numbers of Tcm cells followed by over-differentiation of CD4^+^ and CD8^+^ Tcm cells and high numbers of Tem cells followed by imbalance of CD4^+^ Tem/CD8^+^ Tem cells. It was crucial that these phenomena were reversed by SSP treatment. These results suggested that the therapeutic effect of SSP in DSS-induced colitis was potentially realized by regulating the balance of memory T cells and their subsets.

After acute infection or antigen clearance, the differentiation of memory T cells no longer depends on the classical MHC-II pathway of antigen recognition, but mainly on intracellular signals (*e.g.* PI3K/Akt and JAK/STAT signaling pathways) and inflammatory mediators (*e.g.* IL-2, IL-7, IL-10, IL-12, and IL-15) (Liu and Wu, 2010). Recent evidence has shown that the PI3K/Akt/mTOR and p38 mitogen-activated protein kinase signaling pathways play a key role in the metabolism, differentiation, function, and stability of memory T cells (Carmen Herrero-Sanchez et al., 2016; Huang et al., 2018; Pompura and Dominguez-Villar, 2018). It is also suggested that the regulation of PI3K/Akt signaling has a therapeutic potential in chronic inflammatory diseases mediated by memory T cells.

The PI3K/Akt signaling pathway exists widely in all mammalian cells. It has a profound impact on cell proliferation, survival, differentiation, migration, and metabolism. Under normal circumstances, the PI3K/Akt signaling pathway is strictly controlled by extracellular growth signals, amino acids, and glucose. Over-activation of PI3K/Akt can affect genetic and epigenetic, posttranscriptional, posttranslational and posttranslational abnormalities (Danielsen et al., 2015) to induce T-cell mediated autoimmune diseases (Oak and Fruman, 2007). Akt is involved in a large number of important signaling pathway changes through the phosphorylation of serine or threonine to downstream target signal molecules, including mTOR.

**Figure 7:**
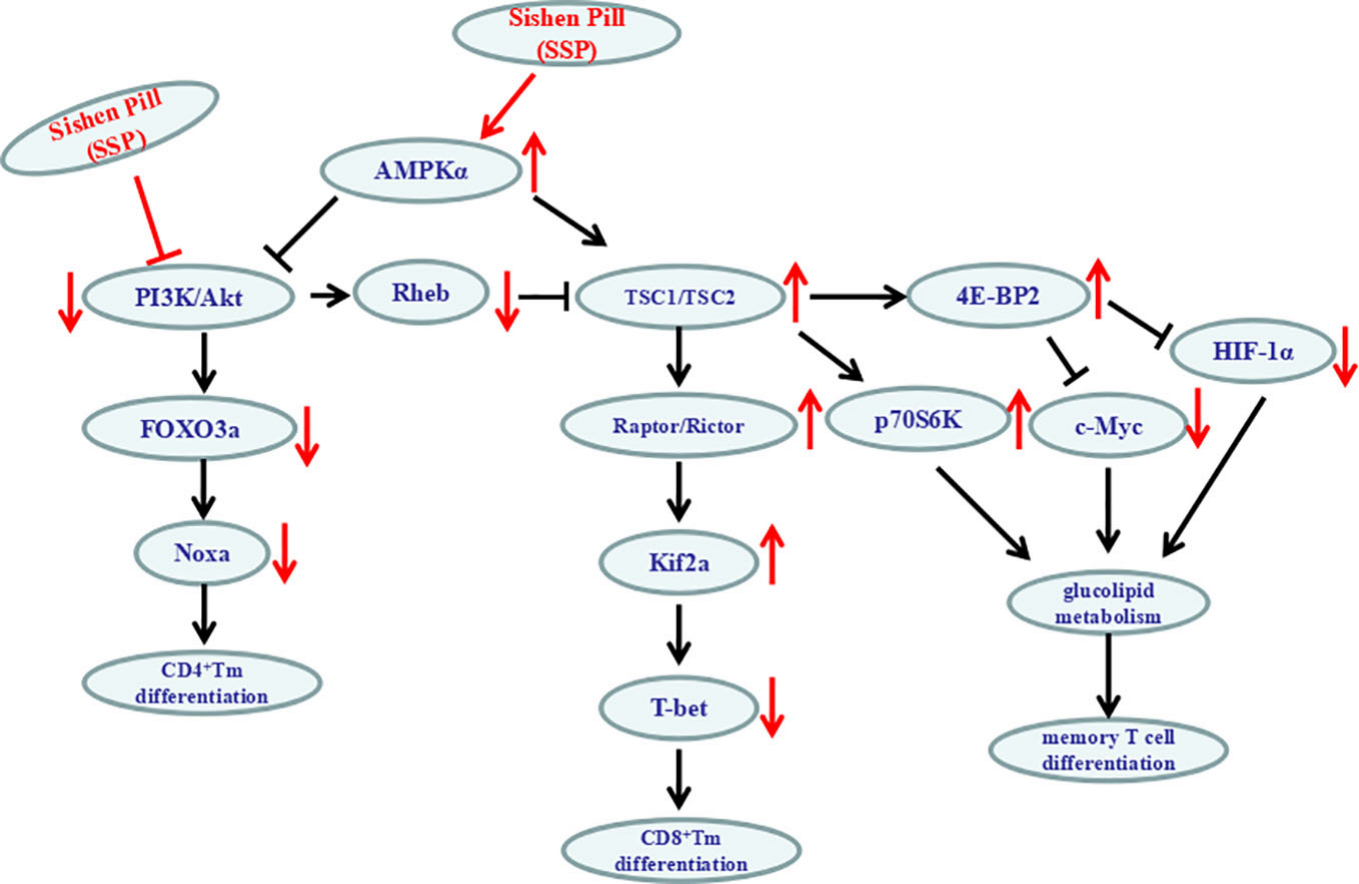
Schematic diagram of SSP regulation of PI3K/Akt signaling pathway.

It is extremely intricate that the PI3K/Akt/mTOR signaling pathway regulates the differentiation of memory T cell (**Figure 7**). In lymphocytes, as a pivotal role for the phosphorylation and dephosphorylation of 4E-BP in the regulation of antigen-driven cell growth and proliferation, the abundance of the 4E-BP2 was much greater than that of 4E-BP1; antigenic recurrent stimulation of memory T cells leads to activation of the rapamycin-sensitive complex mTORC1; mTORC1 phosphorylates 4E-BP2 at the Thr^37^ and Thr^46^ phosphoacceptor sites (Abraham, 2016) to inhibit the activation of C-myc and HIF-1 and furthermore improves glucolipid metabolism to influence memory T cell growth, cell cycle progression, and differentiation. So PI3K/Akt-mTORC1–4E-BP2 signaling axis was centrally in the process of the differentiation of memory T cell.

mTORC1 activation by Akt phosphorylation can produce negative regulatory factors TSC2 and PRAS40. Activated Akt can also degrade TSC1/TSC2 complex and prevent degradation of Rheb/GTP (positive modulin of mTORC1) so as to maintain the inhibitory function of mTORC1. mTORC1 can inhibit the activity of Kif2 and upregulate expression of eomesodermin (Laplante and Sabatini, 2012; Crawley et al., 2014). mTORC2 can directly phosphorylate Akt (Ser473) and then phosphorylate FOXO3a. However, unphosphorylated FOXO3a can induce *IL-7Ra* and *Kif2* gene expression. Furthermore, expression of adhesion molecules or chemokine receptors such as CD62L and CCR7 on the surface of immune memory T cells is controlled (Dong et al., 2012). Phosphorylation of Akt can inhibit the transcription factor FOXO to restrict the number of memory CD8^+^ T cells by inhibiting the accumulation of precursor Tem cells (Hedrick et al., 2012). So, the number of memory CD8^+^ T cells in the spleen of FOXO3-deficient mice increased significantly (Sullivan et al., 2012). AMPK can monitor and maintain energy homeostasis and form in tandem upstream of the TSC1/2/mTOR pathway (Liang and Mills, 2013). Due to the lack of antigen stimulation and the lowest level of inflammatory cytokines, the subsequent decrease in the level of glucose in the cytoplasm leads to the activation of AMPK. AMPK*α*1 acts as a metabolic switch in effector T cells. It can inhibit mTORC1 through the TSC1/2/Rheb pathway, change the metabolic state of effector T cells to static state, and promote differentiation of memory CD8^+^ T cells (Araki and Ahmed, 2013).

Inflammatory mediators (*e.g.* IL-2, IL-7, IL-10, IL-12, and IL-15) are important in the differentiation of memory T cells induced by the PI3K/Akt signaling pathway. IL-12 can promote the transformation of CD8^+^ T cells into CD8^+^ Tem cells by upregulating T-bet and downregulating eomesodermin (Wang et al., 2017). IL-7 can induce and maintain differentiation, survival and proliferation of CD4^+^ Tem cells *via* the PI3K/Akt signaling pathway (Lu et al., 2014). IL-7 combined with IL-15 can induce a large number of differentiated CD8^+^ T cells or maintain IL-7Rα^low^CCR7^−^ memory CD8^+^ T cells to establish antigen-specific immune memory (Kim et al., 2007; Saito et al., 2016). IL-2 can also inhibit IL-7-mediated CD4^+^ Tem cell production through the PI3K/Akt signaling pathway (Lu et al., 2014). It is known that IL-10 is an important anti-inflammatory factor, which plays a crucial role in the process of transform of T cell. In terms of intracellular signaling, including T-bet, eomesodermin, Id2, Blimp-1, and other transcription factors, T-bet and eomesodermin deficiency results in a serious decrease in the number of Tem/Tcm cells. The synergistic effect of T-bet and eomesodermin could promote the phenotype of CD8^+^ Tem/Tcm cells (Li et al., 2013). The proliferation of CD8^+^ Tcm cells induced by IL-2-dependent PI3K/Akt pathway is also related to the rapid and selective expression of eomesodermin (Cho et al., 2013). Deletion of Id2 can promote apoptosis of CD8^+^ T cells and reduce memory cell formation (Cannarile et al., 2006), which is related to Akt-mediated Ezh2 phosphorylation (He et al., 2017). Blimp-1 regulates the formation of T cell memory negatively, and its deletion can enhance survival of memory cells (Ji et al., 2011). IL-7 can downregulate the expression of proapoptotic protein Bim to induce cell survival and proliferation (Jacobs et al., 2010). The role of IL-7R*γ*c signaling in maintaining the long-term survival of memory CD4^+^ T cells is related to the phosphorylation of FOXO3a and can be induced by Akt activation to promote expression of FasL and Bim (Juffroy et al., 2010) and affect the survival of CD4^+^ memory T cells by inhibiting the expression of *Noxa* gene (Schaller et al., 2010; Oh et al., 2012). Therefore, the PI3K/Akt signaling pathway has multitudinous routes to control the differentiation of memory T cells.

According to the above analyses, it is not difficultly found that the Tcm quantity was decreased, while the Tem was increased after the mice were induced by DSS. We think that the antigenic recurrent stimulation was led to transform Tcm to Tem, which included extraneous or endogenous antigenic, disorder gut microbiota and dextran dextran (DSS). These results caused a downregulated Tcm level and an upregulated Tem quantity, and finally improved the levels of CD4^+^ Tcm and CD4^+^ Tem, and inhibited CD8^+^ Tcm and CD8^+^ Tem by activating the PI3K/Akt signaling pathway. The unbalance of the two kinds of memory T cell created a beneficial environment to form the unbalance between the CD4^+^ T cell and CD8^+^ T cell which induced highly-expressed proinflammatory factors (*e.g.* IL-2, IL-7, IL-12, and IL-15) and lower-level anti-inflammatory factors (*e.g.* IL-10) and then led to inflammatory colonic injury and ulceration, which finally caused spontaneous recurrent seizures of IBD. Meanwhile, after DSS-induced colitis mice were treated with SSP, the whole level of Tcm was recovered and Tem was reduced. Synchronously, these numbers of CD4^+^ Tcm and CD4^+^ Tem and the tendencies of CD8^+^ Tcm and CD8^+^ Tem were reversed. We speculated that SSP potentially changed the microenvironment of colon mucosa or some signaling pathway (as PI3K/Akt signal) or rectified the gut microbiota to improve the distribution and subpopulation of immunologic memory T cells. So SSP inhibited the morbigenous memory T cells and improved the advantageous memory T cells to maintain the well-balance of immunological memory T cell. Multitudinous pieces of evidence in the present study have shown that SSP effectively treated DSS-induced colitis, which was realized by regulating the differentiation of memory T cells. However, what is its mechanism?

In the present study, after SSP treatment of DSS-induced colitis for 7 days, PI3K and p-Akt proteins were inhibited and p-AMPK*α* was activated, which suggested that the therapeutic effect of SSP on experimental colitis was closely related to the PI3K/Akt signaling pathway. Many proteins in the PI3K/Akt signaling pathway showed prominent changes in three areas. First, as the main direction, SSP significantly inhibited PI3K and p-Akt activation and downregulated Rheb expression to promote downstream protein activity (TSC1, TSC2, Raptor, Rictor and Kif2a) and suppress T-bet expression and finally regulated differentiation of CD8^+^ memory T cells. Second, SSP significantly activated p-AMPK*α* in DSS-induced colitis. As the center of energy generation, p-AMPK*α* can directly inhibit activation of the PI3K/Akt signaling pathway and promote formation of TSC1/TSC2 to increase 4E-BP2 and p70SK6 phosphorylation to regulate glucolipid metabolism of memory T cells by *C-myc* and *HIF-1α* genes. Finally, SSP inhibited expression of FOXO3a and Noxa proteins to control the degree of differentiation of CD4^+^ memory T cells by decreasing the levels of PI3K and p-Akt activation. It is worth emphasizing that the regulated state of memory T cells and inhibited activation of the PI3K/Akt signaling pathway were synchronously found when SSP alleviated the pathological colonic injury induced by DSS.

Many previous studies have reported that SSP and its effective components can inactivate the PI3K/Akt signaling pathway in other diseases treated by SSP. It is reported that the main effective components of SSP include rutaecarpine, evodiamine, schisandrin B, and psoralen. Evodiamine significantly induces the responses of A375-S2 cell death by PI3K/Akt/caspase and Fas-L/NF-κB signaling pathways (Wang et al., 2010). Rutaecarpine promotes expression of anti-inflammatory cytokines by regulating the IRS-1/PI3K/Akt pathway in the liver (Nie et al., 2016). Schisandrin B significantly increases expression of PI3K, Akt, and p-Akt in cancerous intestinal tissues of patients with colon cancer (Xu et al., 2012; Dai et al., 2018). These findings are a favorable foundation to explore the mechanism of SSP treatment of IBD *via* the PI3K/Akt signaling pathway.

In summary, our data indicated that SSP effectively controls Tem cells in the peripheral blood to relieve experimental colitis induced by DSS, which were potentially related with inhibiting the PI3K/Akt signaling pathway. Of course, we clearly recognized that we should isolate Tcm and Tem cells from the tissue and then use Sishen Pill suspension to treat their cells, and finally extract the Tcm and Tem cell suspension to analyze the activation of PI3K/AKT signal. Or specific Tcm and Tem cell models were prepared by gene knockout and silencing technology and then intervened with Sishen Pill so as to explore the possible mechanism of Sishen Pill regulating immune memory T cells in the treatment of IBD from PI3K/AKT signaling pathway *in vivo* and *in vitro*. However, this is a large and systematic project, and our group has listed it in the next research work as a top-priority.

## Data Availability Statement

All datasets generated for this study are included in the article/supplementary material.

## Ethics Statement

The animal study was reviewed and approved by Institutional Animal Care and Use Committee of Jiangxi University of Traditional Chinese Medicine.

## Author Contributions

Conceived and designed the experiments: D-YL and H-MZ. Performed the experiments: WG, H-YW, H-MZ, X-KL, Y-BZ, JL. Contributed reagents/materials/analytical tools: Z-YZ, D-YL, and H-MZ. Analyzed the data: D-YL, and H-YW. Wrote the paper: WG, H-MZ, and D-YL.

## Funding

This research was supported in part by the National Natural Science Foundation of China (No. 81803988, 8180792, 81760838, 81760808 and 81460679), Natural Science Foundation of Jiangxi Province (No. 20192ACB20015, 20192BAB215050, and 20181BAB205082), Education Department of Jiangxi Province (No. GJJ171542 and GJJ180683), and 1050 Young talents project (No. 1141900603) and first-class subjects starting funds (No. JXSYLXK-ZHYI022, JXSYLXK-ZHYAO132, and JXSYLXK-ZHYAO108) of Jiangxi University of Traditional Chinese Medicine.

## Conflict of Interest

The authors declare that the research was conducted in the absence of any commercial or financial relationships that could be construed as a potential conflict of interest.
